# Phenolic Compounds and Antioxidant Activities of *Liriope muscari*

**DOI:** 10.3390/molecules17021797

**Published:** 2012-02-10

**Authors:** Wen Jie Li, Xian Long Cheng, Jing Liu, Rui Chao Lin, Gang Li Wang, Shu Shan Du, Zhi Long Liu

**Affiliations:** 1 National Institutes for Food and Drug Control, Beijing 100050, China; Email: lwj115@163.com (W.J.L.); 2 State Key Laboratory of Earth Surface Processes and Resource Ecology, Beijing Normal University, Beijing 100875, China; 3 Department of Entomology, China Agricultural University, Beijing 100193, China

**Keywords:** *Liriope muscari*, Liliaceae, antioxidant activity, homoisoflavone

## Abstract

Five phenolic compounds, namely *N*-*trans*-coumaroyltyramine (**1**), *N*-*trans*-feruloyltyramine (**2**), *N*-*trans*-feruloyloctopamine (**3**), 5,7-dihydroxy-8-methoxyflavone (**4**) and (3*S*)3,5,4′-trihydroxy-7-methoxy-6-methylhomoisoflavanone (**5**), were isolated from the fibrous roots of *Liriope muscari* (Liliaceae). Compounds **2**–**5** were isolated for the first time from the *Liriope* genus. Their *in vitro* antioxidant activities were assessed by the DPPH and ABTS scavenging methods with microplate assays. The structure-activity relationships of compounds **1**–**3** are discussed.

## 1. Introduction

Antioxidant activity usually means the ability of a compound to delay, inhibit, or prevent the oxidation of oxidizable materials by scavenging free radicals and reducing oxidative stress [1]. Antioxidants can scavenge ROS (reactive oxygen species) to protect the cells from damage caused by the latter. At present, the most commonly used antioxidants include vitamin C (VC), vitamin E, butylated hydroxyanisole (BHA), butylated hydroxytoluene (BHT), propyl gallate and *tert*-butyl hydroquinone. However, some chemically synthesized antioxidants like BHA and BHT are now being restricted by legislation because of doubts over their possible toxic and carcinogenic effects [2]. Therefore, there is a growing interest in finding antioxidants from natural sources [3,4]. Assays based on the use of 1,1-diphenyl-2-picryl-hydrazyl (DPPH) and 2,2′-azino-bis(3-ethylbenzthiazoline-6-sulfonic acid) (ABTS) radicals are among the most commonly used spectrophotometric methods for determination of the antioxidant capacity of foods, beverages, plant extracts and pure compounds due to the simple, rapid, sensitive, and reproducible procedures involved [5,6]. Phenolic compounds usually possess different antioxidant activity potentials because of their phenolic hydroxy groups which can act as a hydrogen or electron donor [7]. Phenolic acids, flavonoids and tannins are well-known potential natural antioxidants [1]. The hunt for effective and safe antioxidants from natural products is considered to be a shortcut [8].

**Figure 1:**
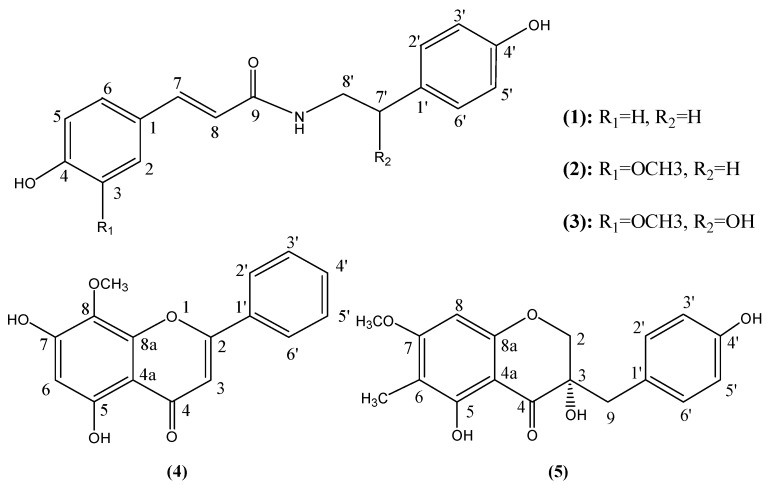
Structures of compounds isolated from *L. muscari.*

*Liriope muscari *(Decne.) Bailey (Liliaceae) is locally known in China as *duantingshanmaidong.* In China, the roots of this species are used locally as a substitute for Radix Ophiopogonis (*maidong* in Chinese) [9], especially in Fujian Province. *Maidong* is a traditional herbal medicine widely used in China as a tonic agent. Modern pharmacological investigations suggest that *maidong* also has an positive effect on various inflammation-related diseases [10]. Previous studies indicated that the main components in *L. muscari* include polysaccharides and steroidal glycosides [11,12,13]. In this paper, five phenolic compounds (Figure 1), including three amides [*N*-*trans*-coumaroyltyramine (**1**), *N*-*trans*-feruloyltyramine (**2**) and *N*-*trans*-feruloyloctopamine (**3**)], one flavone [5,7-dihydroxy-8-methoxy-flavone (**4**)] and one homoisoflavanone [(3*S*)-3,5,4′-trihydroxy-7-methoxy-6-methylhomoiso-flavonone (**5**)] were isolated from *L. muscari*. Compounds **2**–**5** were isolated for the first time from the *Liriope *genus. 

To our knowledge, this is the first time that the phenolic components of *L. muscari *have been studied. Their *in vitro* antioxidant activities were assessed by the DPPH and ABTS scavenging method. *N*-*trans*-feruloyltyramine (IC_50_ 28.7, 8.2 μg/mL) and *N*-*trans*-feruloyloctopamine (IC_50_ 14.4, 7.6 μg/mL) showed potential antioxidant activities.

## 2. Results and Discussion

### 2.1. Isolation and Characterization of Compounds ***1**–**5***

The compounds were isolated using silica gel and Sephadex LH-20 gel column chromatography from 80% ethanol extract of *L. muscari*. The structures of compounds **1**–**4** were characterized by examination of their ESI-MS, NMR (1H- and 13C-) data and comparison with literature reports.

**Figure 2:**
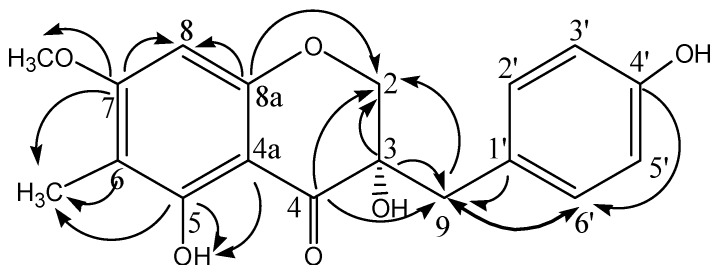
Key HMBC correlations of compound **5**.

Compound **5** was first isolated in 1985 from *Ophiopogonis* [14] (Liliaceae), a closely linked genus that can be easily confused with *Liriope*. In the original paper, the planar structure of compound **5** was identified by comparing the ^1^H-NMR data with that of (3*S*)-3,5,7-trihydroxy-4′-methoxy homoisoflavonone (eucomol, a typical homoisoflavonone isolated from *Eucomis bicolor* BAK. (Liliaceae) [15,16]). In our study, the structure was further confirmed using ^13^C-NMR, APT and 2D-NMR techniques, including ^1^H-^1^HCOSY, HSQC, HMBC (Figure 2). The configuration at C-3 was determined to be (*S*), similar to that of eucomol, based on the positive sign of its specific rotation [16,17]. For a long time, it was believed that there were no homoisoflavones in *Liriope*, so this is the first time a homoisoflavone has been isolated from the *Liriope* genus.

### 2.2. *In Vitro* Antioxidant Activity

#### 2.2.1. DPPH Scavenging Activity

The 1,1-diphenyl-2-picrylhydrazyl radical (DPPH), which possesses an unpaired electron and exhibits a stable violet color in methanol solution (peak absorbance at 517 nm), is commonly used as a reagent for evaluation of the free radical scavenging activity of antioxidants [18]. The DPPH assay is based on the reduction of DPPH in methanol solution in the presence of a hydrogen-donating antioxidant due to the formation of the non-radical form (DPPH-H) in the reaction [19].

**Figure 3:**
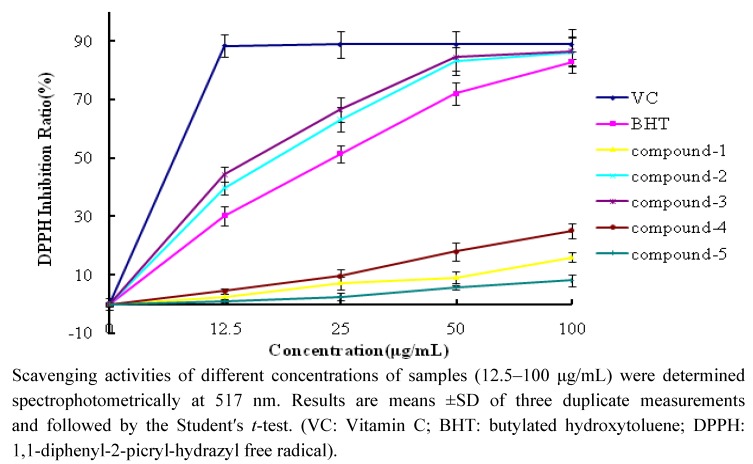
DPPHscavenging activity of compounds **1**–**5** and reference antioxidants.

Figure 3 shows the DPPH scavenging activities of compounds **1**–**5** and reference antioxidants at different concentrations (12.5–100 μg/mL). The test compounds exhibited different DPPH scavenging activities in a concentration-dependent manner. The scavenging effects of compounds **1**–**5** and reference antioxidants on DPPH decreased in the following order: VC > compound **3** > compound **2** > BHT > compound **4** > compound **1** > compound **5**. The inhibition ratios at a concentration of 25 μg/mL are listed in Table 1. Compounds **2** and 3 exhibited effective radical scavenging activity while compounds **1**, **4**, **5** showed very weak activity.

**Table 1:** The DPPH and ABTS inhibition ratio at the concentration of 25 μg/mL.

	DPPH Inhibition Ratio (%)	ABTS Inhibition Ratio (%)
Compound **1**	7.2 ± 0.6	32.7 ± 1.6
Compound **2**	63.2 ± 3.6	75.9 ± 2.0
Compound **3**	66.6 ± 2.3	70.0 ± 3.2
Compound **4**	9.6 ± 1.2	24.3 ± 1.9
Compound **5**	2.6 ± 0.5	16.8 ± 1.7
VC	88.9 ± 4.5	97.4 ± 5.0
BHT	51.5 ± 3.1	95.1 ± 5.3

#### 2.2.2. ABTS Scavenging Activity

In this assay, the 2,2′-azino-bis(3-ethylbenzthiazoline-6-sulfonic acid) (ABTS) radical, which has a peak absorbance at 734 nm, should be preformed by mixing ABTS and potassium persulfate (K_2_S_2_O_8_). When antioxidants were added, the ABTS radical, which has a blue-green color, is reduced to ABTS (no color). Different decoloration abilities indicate different ABTS scavenging activities [20].

**Figure 4:**
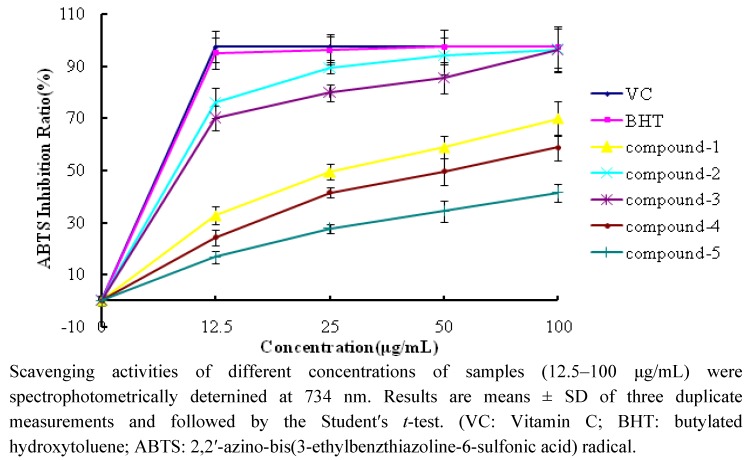
ABTS scavenging activity of compounds **1**–**5** and reference antioxidants.

Figure 4 shows the ABTS scavenging abilities of compounds **1**–**5** and reference standards. The test compounds also exhibited different radical scavenging activities in a concentration-dependent manner like in the DPPH assay. The scavenging effects of compounds **1**–**5** and reference antioxidants on ABTS**^·+^** decreased in the following order: VC ≈ BHT > compound **2** > compound **3** > compound **1** > compound **4** > compound **5**. The inhibition ratios at concentration of 25 μg/mL are listed in Table 1. Compounds **2** and **3** exhibited effective radical scavenging activity, while compounds **1**, **4** and **5** showed relatively weak activity.

In both methods, compounds **2** [21] and 3 [22] showed potential activity, while compounds **1** [23,24] and **4** [25] showed very weak antioxidant activity, which is consistent with the reported results. Furthermore, for compound **4**, quantitative structure-activity relationship analysis [26] also suggested it would not show effective activity because of the absence of 3-OH and *o*-dihydroxy structure in the B ring, and less free -OH groups (only two) in the structure, which are required for high antioxidant activity. As for compound **5**, its antioxidant activity has been previously evaluated using an on-line HPLC-DAD-CL method based on hydrogen peroxide elimination [27]. However, the results cannot be compared with our data due to the absence of any mention of the concentration used in that study. 

**Figure 5:**
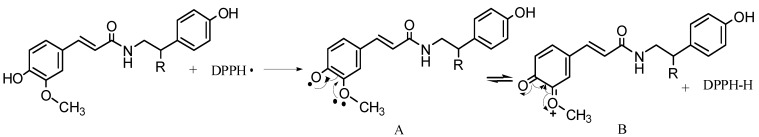
The proposed reaction mechanism between DPPH**^·^** and compounds **1**–**3**.

It is interesting to investigate the structure-activity relationship for compounds **1**–**3**. These compounds have similar structures, but very different activities. Comparing their structures (Figure 1), the main differences are the substituents at C-3 (R_1_) and C-7′ (R_2_). Comparing the structures and activities of compounds **2** and **3** (*p* > 0.05, 25 μg/mL) it is inferred that the presence of methyl group at C-7′ seems to have some, but little influence on antioxidant activity. By comparing compounds **1** and **2** (*p* < 0.05, 25 μg/mL), it is inferred that the presence of methyl group at C-3 is the key factor that affects the activities, therefore, a radical scavenging activity mechanism represented by the reaction shown in Figure 5 is proposed, using DPPH as an example. Compounds **2** and **3** have two resonance structures A and B stabilizing the product, thereby exhibiting superior antioxidant activity than compound **1**. As HPLC evaluation (Experimental section) indicates a relative purity between 90.1% and 96.7% for compounds **1**–**3**, potential synergistic effects from minor impurities could also contribute to the observed activity.

## 3. Experimental

### 3.1. General

^1^H- and ^13^C-NMR spectra were recorded on Bruker Avance DRX 500 instrument using DMSO-*d_6_* or CDCl_3 _as solvent with TMS as internal standard. An Agilent 6320 Ion TRAP LC/MS was employed for MS analysis. The specific rotation was recorded on AUTOPOL IV Automatic Polarimeter (Rudolph, Hackettstown, NJ, USA). For the microplate assay, a SpectraMax 190 Absorbance Microplate Reader (Molecular Devices, Sunnyvale, CA, USA) and 96 Well Cell Culture Cluster (Costar, Corning, NY, USA) were used. 1,1-diphenyl-2-picrylhydrazyl (DPPH) and 2,2′-azino-bis(3-ethylbenzthiazoline-6-sulfonic acid) (ABTS) were purchased from Sigma (Sigma-Aldrich GmbH, Stenheim, Germany). Sephadex LH-20 was purchased from Amersham Pharmacia Biotech AB (Uppsala, Sweden). Polyamide resin (100–200 mesh) was purchased from BeiJingZhongXiYuanDa Technical Co. Ltd. (Beijing, China). Silica gel (160–200 mesh, 200–300 mesh) for column chromatography was purchased from Qingdao Marine Chemical Plant (Shandong Province, China). All other chemicals were of analytical reagent grade and used without any further purification.

### 3.2. Plant Material

Fresh fibrous roots of *L. muscari *were collected from Quanzhou City, Fujian Province, China, in May 2010. The species was identified by Dr. Zhang. J. (National Institutes for Food and Drug Control, NIFDC for short). The voucher specimens were deposited at the herbarium of NIFDC. The roots were air-dried and ground to a powder using a grinding mill (Retsch Muhle, Haan, Germany).

### 3.3. Compound Isolation

In this part, there were mainly two procedures including enrichment and isolation. For the enrichment of phenolic compounds, polyamide resin was used. Polyamide is a commonly used stationary phase for the isolation of phenolic compounds. The enriching mechanism is based on the adsorption power from hydrogen bonds between carbonyl groups of the polyamide and the phenolic hydroxyl groups of target compounds or the polyamide amides and carbonyl groups of fatty acids, *etc.*, The strength of adsorption is dependent on the number of phenolic hydroxyls exposed and their position in the molecule [28] and the power is strongest in water, while getting weaker when the ethanol concentration in the mobile phase is increased.

The detailed procedures are as follows: the powder (2 kg) was extracted three times with 80% hot ethanol (1 L), for 1 h each time. The extracts were concentrated to afford a syrup (1 kg), which was dissolved in 10% ethanol (4 L). Polyamide (1 kg) was added into the solution and stirred about 1 h to make sure the phenolic compounds were adsorbed on the polyamide to some extent. Then the polyamide was centrifuged to dryness (1,000× g, 10 min). Fresh water was used to rinse the polyamide several times till the water was nearly colorless. Then 95% ethanol was used to rinse the polyamide and the solution was collected. The ethanol solution was evaporated to dryness under reduced pressure to afford a solid residue (30 g). The solid residue was chromatographed over a silica gel (160–200 mesh) column (45 × 6.0 cm i.d.) with CHCl_3_/MeOH (20:1 to 8:1) to afford 30 fractions (F01–F30). Fraction F03 (2.2 g) was subjected to Sephadex LH-20 column (120 × 2.5 cm i.d.) chromatography with CHCl_3_/MeOH (10:1) to afford 11 subfractions (F0301–F0311). Then fraction F0308 (69 mg) was chromatographed over a silica gel column (200–300 mesh, 30 × 2.0 cm i.d.) with petroleum/EtOAc (P/E 6:1 to 3:1) to afford compound **4** (10 mg, crystal, P/E 6:1) and compound **5** (8 mg, crystal, P/E 3:1). The purities were 96.3% and 97.2%, respectively (HPLC, 254 nm with PDA detector). As for the isolation of compounds **1**–**3**, fractions F10–F11 (500 mg), fractions F06-F08 (800 mg) and fractions F12–F14 (500 mg) were separated on a Sephadex LH-20 gel column (120 × 2.0 cm i.d.) with MeOH to afford 11 subfractions (F1001–F1011), 19 subfractions (F0601–F0619), and 17 subfractions (F1201–F1217), respectively. Then subfractions F1006 (40 mg), F0609 (100 mg), F1205 (120 mg) were chromatographed over a silica gel column (200–300 mesh, 30 × 2.0 cm i.d.) with P/E (1:1), P/E (3:2 to 1:1), P/E (1:1 to 1:2) to afford compounds **1** (10 mg), **2** (8 mg) and **3** (15 mg). The purity of compounds **1–3** was 96.6%, 90.1% and 91.1%, respectively (HPLC, 254 nm with PDA detector).

*N-trans-coumaroyltyramine *(**1**). White powder (petroleum/acetic ether). R_f_ 0.65 (acetic ether) ESI-MS: *m/z* 284 [M+H]^+^. C_17_H_17_NO_3_. ^1^H-NMR (DMSO-*d_6_*, 500 MHz) δ: 9.36 (-OH), 8.11 (1H, t, 5.0 Hz, -NH), 7.39 (2H, d, 8.5 Hz, H-2, 6), 7.30 (1H, d, 15.5 Hz, H-7), 7.01 (2H, d, 8.0 Hz, H-2′, 6′), 6.78 (2H, d, 8.5 Hz, H-3, 5), 6.67 (2H, d, 8.0 Hz, H-3′, 5′), 6.38 (1H, 15.5 Hz, H-8), 3.31 (2H, m, H-8′), 2.64 (2H, t, 7.5 Hz, H-7′). The ^1^H- and ^13^C-NMR (Table 2) spectral data are consistent with published data [29,30].

*N-trans-feruloyltyramine *(**2**). Colourless oil (petroleum/acetic ether). R_f_ 0.62 (acetic ether) ESI-MS: *m/z* 314 [M+H]^+^. C_18_H_19_NO_4_. ^1^H-NMR (DMSO*-d_6_*, 500 MHz) δ: 9.46, 9.21 (C_4_-OH, C_4′_-OH), 8.01 (1H, t, 5.0 Hz, -NH), 7.31 (1H, d, 16.0 Hz, H-7), 7.12 (1H, s, H-2), 7.01 (2H, d, 8.0 Hz, H-2′, 6′), 6.98 (1H, m, H-6), 6.78 (1H, d, 8.2 Hz, H-5), 6.68 (2H, d, 8.0 Hz, H-3′, 5′), 6.43 (1H, 15.5 Hz, H-8), 3.80 (3H, s, -OCH_3_), 3.33 (2H, m, H-8′), 2.64 (2H, t, 7.5 Hz, H-7′). The ^1^H- and ^13^C-NMR (Table 2) spectral data are consistent with published data [31,32].

*N-trans-feruloyloctopamine* (**3**). Colourless oil (petroleum/acetic ether). R_f_ 0.47 (acetic ether) ESI-MS: *m/z *330 [M+H]^+^. C_18_H_19_NO_5_. ^1^H-NMR (DMSO-*d_6_*, 500 MHz) δ: 9.47, 9.32 (C_4_-OH, C_4′_-OH), 7.96 (1H, t, 5.5 Hz, -NH), 7.31 (1H, d, 15.5 Hz, H-7), 7.15 (2H, d, 8.5 Hz, H-2′, 6′), 7.12 (1H, s, H-2), 6.98 (1H, d, 7.5 Hz, H-6), 6.79 (1H, d, 8.5 Hz, H-5), 6.72 (2H, d, 8.5 Hz, H-3′, 5′), 6.55 (1H, 15.5 Hz, H-8), 4.54 (1H, m, H-7′), 3.80 (3H, s, -OCH_3_), 3.38, 3.18 (2H, m, H-8′). The ^1^H- and ^13^C-NMR (Table 2) spectral data are consistent with published data [32,33].

*5,7-Dihydroxy-8-methoxyflavone* (**4**). Yellow needle crystal (petroleum/acetic ether). R_f_ 0.40 (P/E 2:1) ESI-MS: *m/z* 283 [M−H]^−^. C_16_H_12_O_5_. ^1^H-NMR (DMSO-*d_6_*, 500 MHz) δ:12.52 (1H, s, 5-OH), 10.87 (1H, s, 7-OH), 8.08 (2H, d, 6.5 Hz, H-2′, 6′), 7.62 (3H, m, H-3′, 4′, 5′), 7.02 (1H, s, H-3), 6.32 (1H, s, H-6), 3.86 (3H, s, -OCH_3_). ^13^C-NMR (DMSO-*d_6_*, 125 MHz) δ: 182.5 (C-4), 163.5 (C-2), 157.8 (C-7), 156.7 (C-5), 150.1 (C-8a), 132.6 (C-4′), 131.3 (C-1′), 129.8 (C-3′, 5′), 128.2 (C-8), 126.8 (C-2′, 6′), 105.5 (C-3), 104.2 (C-4a), 99.6 (C-6), 61.5 (-OCH_3_). The ^1^H- and ^13^C-NMR spectral data are consistent with published data [34,35].

*(3S)-3,5,4′-trihydroxy-7-methoxy-6-methyl homoisoflavonone *(**5**). Colorless needle crystal (CHCl_3_). R_f_ 0.36 (P/E=2:1) (*c*=0.0100, CHCl_3_) ESI-MS: *m/z *329 [M−H]^−^. C_18_H_18_O_6_. ^1^H-NMR (CDCl_3_, 500 MHz) δ: 7.09 (2H, d, 8.0 Hz, H-2′, 6′), 6.78 (2H, d, 8.0 Hz, H-3′, 5′), 6.10 (1H, s, H-8), 4.23 (1H, d, 11.0 Hz, H-2a), 4.06 (1H, d, 11.0 Hz, H-2b), 3.91 (3H, S, -OCH_3_), 2.96 (2H, dd, 14 Hz, 5.0 Hz), 2.06 (3H, s, -CH_3_). ^13^C-NMR (CDCl_3_, 125 MHz) δ: 198.3 (C-4), 166.5 (C-7), 161.1 (C-8a), 160.1 (C-5), 154.9 (C-4′), 131.8 (C-2′,6′), 126.2 (C-1′), 115.3 (C-3′,5′), 106.5 (C-6), 100.2 (C-4a), 91.0 (C-8), 72.3 (C-3), 71.9 (C-2), 56.0 (-OCH_3_), 40.8 (C-9), 6.9 (-CH_3_). The ^1^H-NMR spectral data are consistent with published data [14].

**Table 2:** ^13^C-NMR data of compounds **1**–**3** (DMSO-*d_6_*, 125 MHz).

Position	Compound 1	Compound 2	Compound 3
1	126.3	126.9	126.9
2	129.8	111.2	111.2
3	116.2	148.3	148.3
4	159.2	148.7	148.7
5	116.2	116.1	116.1
6	129.8	122.0	122.0
7	139.3	139.3	139.4
8	118.9	119.5	119.6
9	166.1	165.8	166.0
1′	129.1	130.0	134.5
2′	130.0	129.9	127.6
3′	115.6	115.6	115.2
4′	156.0	156.1	156.9
5′	115.6	115.6	115.2
6′	130.0	129.9	127.6
7′	34.7	34.9	71.6
8′	41.2	41.1	47.5
-OCH_3_		56.0	55.9

### 3.4. Antioxidant Ability

#### 3.4.1. DPPH Assay

In this method, a microplate reader and 96 well plate were used to carry out the determination of the spectral absorption values. This assay is based on the classic method developed by Blois in 1958 [19]. Various forms of this method are widely used [36,37]. Unlike the commonly used methods, which are labor and time-consuming and reagent and sample-wasting, this microplate assay method is much more rapid, sample-saving and environmentally-friendly. In this method, methanolic DPPH solutions (100 μg/mL, 50 μL) were added to samples of different concentration (200 μL, 12.5–100 μg/mL). These solutions were gently mixed and incubated in the dark for 30 min at room temperature. Then the absorbances of the resulting solutions were measured at 517 nm. For preparation of the standard curve, different concentrations of DPPH methanol solutions (5–50 μg/mL) were used. The DPPH concentration (μg/mL) in the reaction medium was calculated from the following calibration curve, determined by linear regression (*r*^2^: 0.9985): 


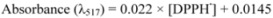


The scavenging capability of test compounds was calculated using the following equation:

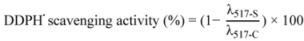

where λ_517-C_ is absorbance of a control with no radical scavenger and λ_517-S_ is absorbance of the remaining DPPH in the presence of scavenger.

#### 3.4.2. ABTS Assay

The ABTS assay was carried out using a method based on the original and classic method developed by Miller in 1993 [38] with some modifications. The ABTS radical should be preformed by reacting equal volumes of 1.1 mg/mL aqueous ABTS and 0.68 mg/mL potassium persulfate (K_2_S_2_O_8_), and then storing in the dark for 6 h at room temperature, as described by Gülçin [6]. Then ABTS**^·+ ^**solutions (50 μL) were added to samples of different concentrations (200 μL, 12.5–100 μg/mL). These solutions were gently mixed and incubated in the dark for 30 min at room temperature. Then the absorbances of the resulting solutions were measured at 734 nm. Different concentrations of ABTSradical solutions (55–220 μg/mL) were used to prepare the standard curve. The ABTS radical concentration (μg/mL) in the reaction medium was calculated from the following calibration curve, determined by linear regression (*r*^2^: 0.9985):


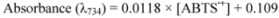


The scavenging capability of test compounds was calculated using the following equation:

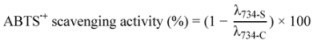

where λ_734-C_ is absorbance of a control with no radical scavenger and λ_734-S_ is absorbance of the remaining ABTS in the presence of scavenger.

## 4. Conclusions

Phenolic components of *L. muscari *were studied for the first time. Three amides, one flavone and one homoisoflavonone were isolated and their antioxidant activities were evaluated using two microplate assay methods. *N*-*trans*-feruloyltyramine (**2**) and *N*-*trans*-feruloyloctopamine (**3**) showed effective activity and the structure-activity relation investigation indicates that the -OCH_3 _group at C-3 affects the antioxidant activity.

## Supplementary Materials

Supplementary materials can be accessed at: http://www.mdpi.com/1420-3049/17/2/1797/s1.
