# Supplementary material for: Phenolic Compounds and Antioxidant Activities of Liriope muscari
Source: Molecules. 2012 Feb 10;17(2):1797–808. doi: 10.3390/molecules17021797 (PMC6268958; doi:10.3390/molecules17021797)
Supplement: Supplementary file 1 [file molecules-17-01797-s001.docx]

*Supplementary Materials*

Phenolic Compounds and Antioxidant Activities of
*Liriope muscari*

**Wen Jie Li ^1^, Xian Long Cheng ^1^, Jing Liu ^1^, Rui Chao Lin ^1,^*, Gang Li Wang ^1,^*, Shu Shan Du ^2^ and Zhi Long Liu ^3^**

^1^ National Institutes for Food and Drug Control, Beijing 100050, China

^2^ State Key Laboratory of Earth Surface Processes and Resource Ecology, Beijing Normal University, Beijing 100875, China

^3^ Department of Entomology, China Agricultural University, Beijing 100193, China

***** Authors to whom correspondence should be addressed; E-Mails: Linrch307@sina.com.cn (R.C.L.); duneer@163.com.cn (G.L.W.); Tel.: +86-10-6709-5307 (R.C.L.); Fax: +86-10-6702-3650 (R.C.L.).

Received: 24 November 2011; in revised form: 24 January 2012 / Accepted: 31 January 2012 /
Published: 10 February 2012

**Figure 1.** ^1^H of compound **5**.

**Figure 2.** ^13^C of compound **5**.

**Figure 3.** APT of compound **5**.

**Figure 4.** ^1^H-^1^H-COSY of compound **5**.

**Figure 5.** **HSQC** of compound **5**.

**Figure 6.** **HMBC** of compound **5**.
